# Coplanar Meta-Surface-Based Substrate-Integrated Waveguide Antennas with Broadband and Low Reflections for K-Band Beam Scanning

**DOI:** 10.3390/s22176353

**Published:** 2022-08-24

**Authors:** Chunli Wang, Dongxing Gao, Likai Liang, Yanling Wang

**Affiliations:** School of Mechanical, Electrical and Information Engineering, Shandong University, Weihai 264209, China

**Keywords:** substrate-integrated waveguide (SIW), mm-Wave antennas, meta-surface, beam scanning, broadband

## Abstract

Four novel substrate-integrated waveguide (SIW) antennas are proposed, in order to obtain K-band beam scanning through the coplanar meta-surfaces of properly devised complementary split-ring resonators. More specifically, coplanar rhombus- and hexagon-shaped meta-surfaces replace the metallized via holes in the traditional SIW structure, achieving low reflection and wide bandwidth, respectively. Another trapezoid-shaped meta-surface is introduced, in order to realize good leaky-wave radiation performance with high-gain beam scanning in both rhombus- and hexagon-shaped SIW components. These designs are further extended to two different mixed types of two-row meta-surfaces, with the rhombus and hexagon structures combined in different orders to enhance the complex SIW transmission lines and antennas, which can simultaneously obtain good reflection and bandwidth with different priority, depending on the arrangement. We explain the performance differences with rhombus and hexagon meta-surfaces through the analysis of relevant equivalent circuit models and extracting the effective medium parameters, and we verify the bandwidths and radiations of four SIW antennas both numerically and experimentally. The maximum gains of the four antennas are 18.1 dBi, 17.0 dBi, 18.8 dBi and 17.1 dBi, where the corresponding relative bandwidths are 10.74%, 19.42%, 14.13% and 18.38%. The maximum simulated radiation efficiency and aperture efficiency of the proposed antennas are 91.20% and 61.12%, respectively. Our approach for generating flexible and selectable tuned electromagnetic fields from SIWs is applicable for the development of mm-Wave antennas or sensors on PCB-integrated platforms for highly directive scanning radiation.

## 1. Introduction

Substrate-integrated waveguides (SIWs) have attracted the attention of various researchers, due to their great capacity for easy integration and good propagation characteristics on printed circuit board (PCB) substrates, where the double-coated copper and two rows of metallized via holes forming the low-profile and closed structure naturally adapt to the PCB board [1]. The integration advantage of SIWs is due to their similarity to the micro-strip transmission line, while avoiding the large radiation loss from the micro-strip structure as the frequency increases [2,3]. On the other hand, the propagating modes of an SIW imitate those of rectangular waveguides, with the desirable features of low return loss, high Q, and large power capacity, while solving the miniaturization compatibility problem of conventional waveguides [4,5]. As mobile communication technology shifts to 5G and 6G bands, PCB-integrated SIWs are expected to become more widely applied in antennas and sensors [6,7,8]. Drilling and metallization techniques become more difficult in mm-Wave bands, due to the reduced volume of the electromagnetic devices. The thinner PCB board is also easily deformed under mechanical pressure, resulting in a deterioration in electromagnetic performance. Meanwhile, the flexible adjustment of bandwidth and radiation is urgently required in antennas or sensors for various complex platforms.

The use of meta-surfaces [9,10,11,12] has been shown to be an efficient way to build novel electromagnetic devices, including high-gain beam redirection [13,14,15] and multi-beam control with enhanced radiations [16,17], where the abrupt phase discontinuities provided by meta-surfaces could offer a straightforward method to manipulate electromagnetic waves. Such a method is also similar to frequency selective surfaces (FSSs), which is one of the periodic interface structures, where the sub-wavelength units enable us to tune the electrical properties, such as amplitude, phase and polarization, of the incoming waves, thus fulfilling the high-gain radiations through reshaping the wavefront simply at surfaces [18,19]. Moreover, the capacity of meta-surfaces of perfectly integrating with PCB platforms simply by using copper cladding has also been demonstrated [20,21,22,23]. On this basis, specifically designed classical complementary split-ring resonators have been proposed to imitate a virtual electric wall in the SIW design and remove conventional metallized via holes [24]. However, in order to design mm-Wave SIW devices with stronger performance and more flexible parameters, it is necessary to further couple the meta-surface and SIW structure. We can achieve the free manipulation of electromagnetic fields and specific adjustment of the bandwidth, reflection coefficient and pattern performance through the introduction of different types of sub-wavelength meta-atom arrays into the desired SIWs [25,26,27,28,29], thus initiating the quest for tangible applications in the SIW transmission line and antenna scenarios.

In this paper, we propose four packs of SIW antennas to obtain beam scanning, low reflection and wide bandwidth performance through arranging series of properly devised coplanar meta-surfaces. Rhombus- and hexagon-shaped meta-units are independently built to simulate virtual electric walls and replace metal via holes, where the rhombic structure has a steeper *S*-curve and numerically better electric wall performance, while the hexagonal structure focuses on realizing a wider frequency band. These different performances are well explained through analysis of relevant circuit models and calculating the normalized impedance. Rhombus and hexagon meta-surface inspired SIW transmission lines are thus established, achieving lower reflection and wider bandwidths, respectively, as expected. By bringing trapezoid-shaped meta-surfaces to the rhombus and hexagon SIW lines, the coplanar meta-surface-based SIW antennas are further established, demonstrating better leaky-wave performance as well as preservation of the original bandwidth parameters. Finally, we combine the rhombus and hexagon structures in different orders to simultaneously obtain good reflection and bandwidth, with different emphasis due to the relative SIW transmission lines. Trapezoid-shaped meta-surfaces are also introduced to these hybrid SIWs, forming two other new coplanar meta-surface-based SIW antennas, which achieve the expected radiations. All four of the proposed antennas have the advantages of more flexibly controlling the reflection coefficient and bandwidth, while also demonstrating good leaky-wave and beam-scanning performances. Our work aims to guide the selection of a specific combination of meta-surfaces to meet the various demands of electromagnetic devices, thus broadening the application scope of mm-Wave PCB-integrated antennas or sensors.

## 2. Meta-Surface-Based SIW Component Design and Analysis

### 2.1. Unit Cell Design

**Figure 1:**
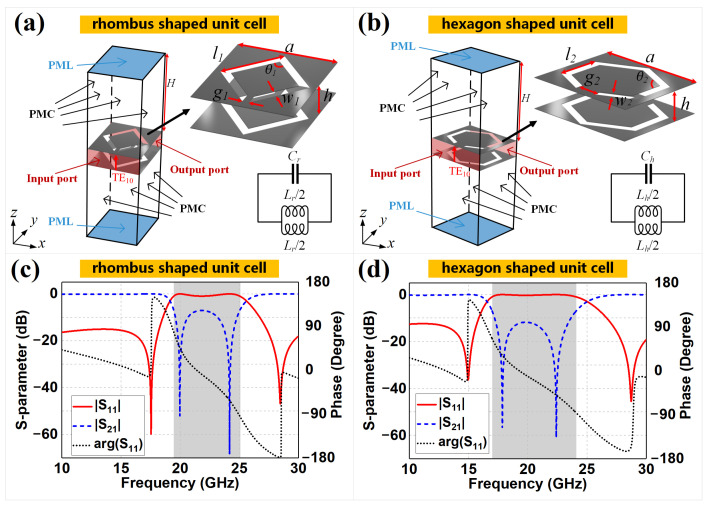
The Floquet model and corresponding equivalent circuits of the (**a**) rhombus- and (**b**) hexagon-shaped unit cells. Each unit has the dimensions of 2.5 mm × 2.5 mm × 0.787 mm and the substrate is chosen as RT5880 material, with relative permittivity of 2.2 and loss tangent of 0.001. Moreover, *a* = 2.5 mm, *h* = 0.787 mm, *H* = 3.75 mm, $l_{1}$ = 1.58 mm, $l_{2}$ = 1.2 mm, $\theta_{1}$ = $90^{\circ }$, $\theta_{2}$ = $120^{\circ }$, $g_{1}$ = 0.16 mm, $g_{2}$ = 0.5 mm, $w_{1}$ = 0.2 mm, and $w_{2}$ = 0.2 mm, based on the optimization results. The *S*-parameter of the (**c**) rhombus- and (**d**) hexagon-shaped unit cells is also provided.

Let us start with the design of unit cells for the rhombus- and hexagon-shaped meta-surfaces around 20 GHz, as shown in Figure 1. The two unit elements share a similar complementary split-ring resonator template, but have different geometric structures and sizes, leading to different *S*-parameter simulations and circuit parameters. Considering a traditional SIW, the metallized via holes are equivalent to small dipoles and, thus, act as an electric wall, fulfilling the surface current loop driven by the ${\mathrm{TE}}_{m0}$ mode flowing along each via [1]. Note that the classical complementary split-ring resonator is capable of imitating the virtual electric wall and keeping the horizontal incoming waves fixed in the closed cavity at specific narrow bands [30,31]. Hence, we modify the geometric structures of classical resonators and rebuild the rhombus- and hexagon-shaped unit cells with different wide-bands around 20 GHz. The unit length *a* is set to 2.5 mm that is $\lambda_{0}/6$ at 20 GHz, which meets the requirements that the periodic composite structure could be applied as sub-wavelength atoms of meta-surfaces at such volume scale and specific frequency [32,33,34,35,36]. Further, the length $l_{1}$ and $l_{2}$ of the rhombus- and hexagon-shaped cells could be determined by their specific geometry and unit length *a*, following the traditional design method of meta-surface units. The rhombus (or hexagon)-shaped slots are etched on the top and bottom PEC planes, where the two slots have the same geometric sizes as listed in the figure caption but opposite opening directions of the gaps, as shown in Figure 1a (or Figure 1b). To verify the blocking performance generated from the coupling effect between the complementary rings of the unit, the *S*-parameter performances are simulated based on the enhanced Floquet mode with a vertically polarized electric field feeding in port 1 and receiving in port 2, where the ports are attached to the front and back of the substrates, which are vertical to the *y*-axis. The symmetrical spaces above and below the substrates are filled with air, with the top and bottom boundaries as a perfectly matched layer (PML), while the remaining boundaries are set as perfect magnetic conductors (PMCs). Periodic boundary conditions are, thus, indirectly enforced, repeating the modeled structure periodically along the *x*- and *y*-axes. The simulated results are shown in Figure 1c,d. We can observe that both the rhombus and hexagon units present sufficiently wide bands, where the $S_{11}$ results higher than −10 dB range from 18.53 to 26.72 GHz and 15.86 to 26.66 GHz, respectively. The −1 dB bandwidth of $S_{11}$ results used for evaluating shielding effects are 19.45 to 25.05 GHz for the rhombus-shaped unit cell and 16.98 to 24.10 GHz for the hexagon-shaped unit cell, as shown in the gray areas. Moreover, the phase component of the $S_{11}$ value is also simulated for further analysis, plotted as black dotted lines in Figure 1c,d. Both the phases of the rhombus- and hexagon-shaped units decrease approximately and linearly within the transmission interval and the shielding interval, while experiencing abrupt value changing near the resonance frequency points. A high $S_{11}$ level corresponds to a low $S_{21}$ value, which means that the unit is equivalent to an electric wall at these frequency ranges, and that the propagating mode through the cell is prohibited. We can also observe that the rhombus unit has a steeper slope near the minimum transmission regions at 19.97 GHz and 24.17 GHz, while the hexagon structure has 31.87% more bandwidth, based on the $S_{11}$ values.

To explain these different resonant properties, we propose the equivalent circuit models of rhombus and hexagon cells, as shown in Figure 1a,b, based on the basic equivalent circuits of classical split-ring resonators [37,38], as well as the duality principle [39,40,41,42]. In particular, the equivalent circuit of the classic double-layer split ring resonator is an LC parallel resonance circuit [24,43,44]. We start with such a classical resonance circuit extracted from a circular ring and change the circuit form to adapt to rhombus and hexagon unit cells using the duality principle and analyzing geometry changes. For the physical structure of the classical circular ring and the proposed rhombus and hexagon rings, the corresponding circuit parameters could be calculated through analyzing the detailed micro-structure of the rings, where the intervals between rings mainly represent capacitance, and the ring with a specific length mainly represents inductance. Therefore, the corresponding capacitance and inductance could be updated through comparing the geometric differences between the classical circular ring and proposed rhombus and hexagon rings. The total capacitance is obtained by calculating the series capacitances $C_{SRR}/2$ of two semi-circular ring loops. As the ring is a double-layer structure, the unit length capacitance of the microstrip line can also be calculated as a series capacitance of two microstrip lines on both layers. That is, the unit length capacitance $C_{m}$ of each side can be expressed as $C_{m}=\sqrt{\varepsilon_{\mathrm{eff}\;}}/c_{0}Z_{0}$, with its substrate height equal to half of the total substrate thickness, where $\varepsilon_{\mathrm{eff}\;}$, $c_{0}$ and $Z_{0}$ are the effective dielectric constant, speed of light and microstrip line characteristic impedance, respectively [45]. The total unit length capacitance is equal to $C_{m}/2$, when considering the series effect of the two layers. Thus, the capacitance value, $C_{SRR}$, can be expressed as $C_{\mathrm{SRR}\;}=C_{m}L_{\mathrm{eff}\;}/2$, where $L_{\mathrm{eff}}$ is the effective perimeter of the ring. The total inductance of the standard circular ring loop can be expressed as $L_{SRR}=u_{0}u_{r}\left(D_{\mathrm{eff}\;}/2\right)\left(\ln\left(8D_{\mathrm{eff}\;}/w\right)-2\right)$, based on [46], where $D_{\mathrm{eff}}$ and *w* represent the effective diameter and wire width of the ring, respectively. On this basis, the complementary split-ring resonators of rhombus and hexagon cells are equivalent to a duality circuit of the LC parallel resonance circuit mentioned above, as the units used here are complementary rings [44,47]. Thus, the converted capacitance $C_{r}$ ($C_{h}$) is calculated by multiplying the original inductance $L_{SRR}$ by a factor of $4\varepsilon_{0}/\mu_{0}$, while the converted inductance $L_{r}$ ($L_{h}$) is equal to the quotient of the original capacitance $C_{SRR}$ and the same factor [38]. The derived formulas are as follows:

(1) $$C_{r}=2\varepsilon_{0}\mu_{r}\frac{l_{1}}{\sqrt{\pi }}\left(\ln\frac{8l_{1}}{\sqrt{\pi }\omega_{1}}-2\right),$$

(2) $$C_{h}=2\varepsilon_{0}\mu_{r}\frac{l_{2}}{\sqrt{\frac{3\sqrt{3}}{2\pi }}}\left(\ln\frac{8l_{2}}{\sqrt{\frac{3\sqrt{3}}{2\pi }}\omega_{2}}-2\right),$$

(3) $$L_{r}=\frac{\mu_{0}}{8\varepsilon_{0}}\frac{\sqrt{\varepsilon_{\mathrm{eff}}}}{c_{0}z_{0}}\left[4\left(l_{1}-\omega_{1}\right)-\sqrt{2}g_{1}\right],$$

(4) $$L_{h}=\frac{\mu_{0}}{8\varepsilon_{0}}\frac{\sqrt{\varepsilon_{\mathrm{eff}}}}{c_{0}z_{0}}\left[6\left(l_{2}-\frac{\omega_{2}}{\sqrt{3}}\right)-\frac{2}{\sqrt{3}}g_{2}\right],$$

where $l_{1}$, $l_{2}$, $w_{1}$, $w_{2}$, $g_{1}$ and $g_{2}$ are labeled in Figure 1 and their values are listed in the corresponding figure caption. The effective diameter and perimeter of the two meta-units, expressed as $D_{\mathrm{eff}}$ and $L_{\mathrm{eff}}$ in the original formulas, can be represented by the specific structural parameters, according to the geometric properties of the rhombus and hexagon unit cells. By comparing the values of the geometric parameters for the rhombus- and hexagon-shaped units shown in Figure 1, we obtained the following expressions: $C_{h}>C_{r}$ and $L_{h}>L_{r}$. Therefore, the hexagon unit design has lower resonant frequencies than the rhombus unit. Considering the electromagnetic response of the periodic composite material at different volume scales and frequency ranges, the analytical model of the electromagnetic structure with constant size may change from the classical medium to the meta-surface, then move to a frequency-selective surface as the frequency increases, where the wavelength gradually becomes comparable to the geometry of electromagnetic devices [32,33,34,35]. This means that the media properties of the rhombus and hexagon unit cells gradually move to the transition region of the meta-surface and frequency-selective surface when the second resonant frequency (i.e., the upper stop-band frequency point) appears as the frequency continues to increase. Compared to the lower-side resonant frequency, the upper-side frequency is less sensitive to the geometric microstructure of the unit cell, leading to the fact that the upper-side frequencies of the rhombus and hexagon meta-units are closer to each other. This phenomenon and Equations (1)–(4) work well together to explain that both of the resonant frequencies of the hexagon unit (17.85 GHz and 22.41 GHz) are lower than those of the rhombus unit (19.97 GHz and 24.17 GHz), and the frequency difference of the lower-side frequencies of the two unit cells is larger than that for the upper-side frequencies. In summary, the hexagon-shaped design has a wider bandwidth than that of the rhombus design.

Another method for analyzing the electromagnetic response of a meta-surface is effective medium theory [48,49], which can be extracted and calculated from the *S*-parameter results. We used the MATLAB software to calculate the normalized transfer impedance $Z_{T}$ of the lower- and upper-side resonant frequencies of the rhombus and hexagon units, which can be applied to quantify the shielding effectiveness of the virtual electric walls. The normalized transfer impedances of the rhombus unit were $7.79\times 10^{-7}$ and $4.63\times 10^{-7}$ at 19.97 GHz and 24.17 GHz, respectively, while the normalized transfer impedances of the hexagon unit were $1.05\times 10^{-5}$ and $7.00\times 10^{-5}$ at 17.85 GHz and 22.41 GHz, respectively. A lower $Z_{T}$ value means that the incoming waves experience a stronger impedance mismatch and amplitude decay, resulting in steeper slopes and narrower bands in the resonant curve. We can conclude that the rhombus design has stronger shielding effectiveness and better virtual electric wall performance near the minimum transmission regions, despite the narrower bands, while the hexagon design has a significantly broader band with acceptably raised $S_{21}$ performance.

### 2.2. SIW Topology

**Figure 2:**
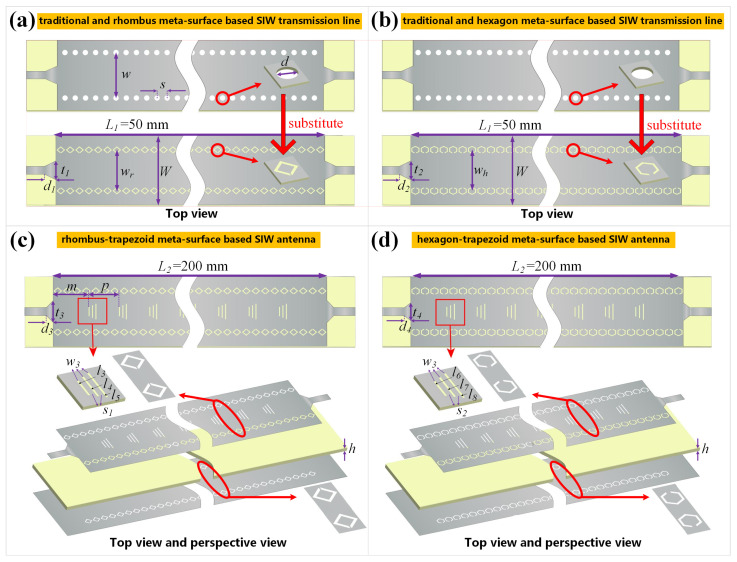
The coplanar (**a**) rhombus and (**b**) hexagon meta-surface-based SIW transmission lines compared with the traditional SIW structure, as well as the coplanar (**c**) rhombus–trapezoid and (**d**) hexagon–trapezoid meta-surface-based SIW leaky-wave antennas. The structural parameters are $L_{1}$ = 50 mm, *W* = 22 mm, $t_{1}$ = 6 mm, $d_{1}$ = 4 mm, $w_{r}$ = 13 mm, $t_{2}$ = 5 mm, $d_{2}$ = 2.5 mm, $w_{h}$ = 13 mm, $L_{2}$ = 200 mm, *m* = 9.4 mm, *p* = 8 mm, $t_{3}$ = 7 mm, $d_{3}$ = 1.8 mm, $t_{4}$ = 5.7 mm, $d_{4}$ = 1.7 mm, and *h* = 0.787 mm. For the trapezoid unit, the geometric sizes are set as: $l_{3}$ = 2.9 mm, $l_{4}$ = 3.9 mm, $l_{5}$ = 4.9 mm, $s_{1}$ = 0.9 mm, $w_{3}$ = 0.2 mm, $l_{6}$ = 3 mm, $l_{7}$ = 4 mm, $l_{8}$ = 5 mm, and $s_{2}$ = 1 mm.

**Figure 3:**
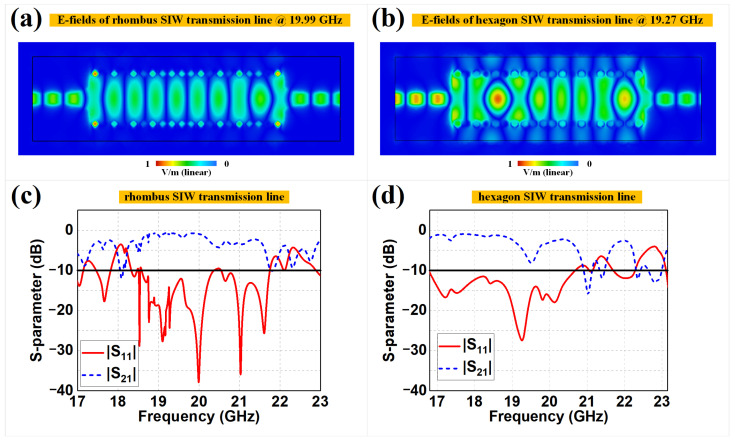
The E-field distributions of the (**a**) rhombus and (**b**) hexagon meta-surface-based SIW transmission lines, as well as the corresponding *S*-parameters of the (**c**) rhombus and (**d**) hexagon meta-surface-based SIW transmission lines.

**Figure 4:**
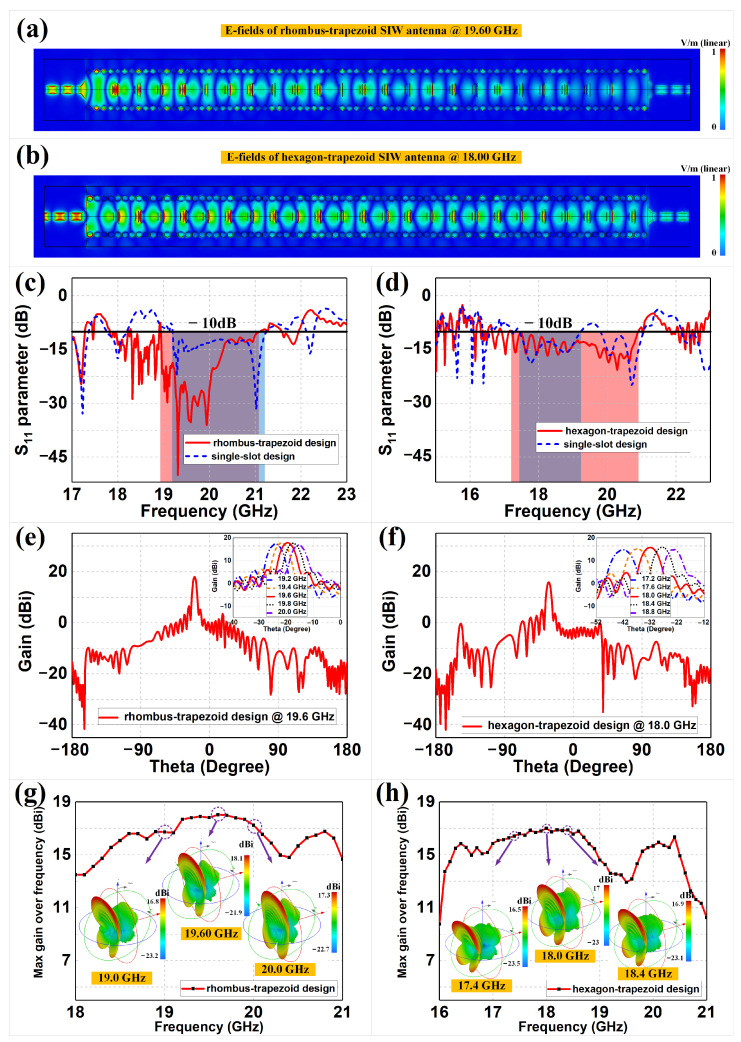
The E-field of the (**a**) rhombus and (**b**) hexagon meta-surface-based SIW leaky-wave antennas, and the corresponding reflection coefficients of the (**c**) rhombus- and (**d**) hexagon-shaped SIW antenna designs with comparison groups. The 2D radiation patterns of the (**e**) rhombus and (**f**) hexagon meta-surface-based SIW leaky-wave antennas, and the corresponding maximum gains and 3D radiations of the (**g**) rhombus- and (**h**) hexagon-shaped designs.

**Figure 5:**
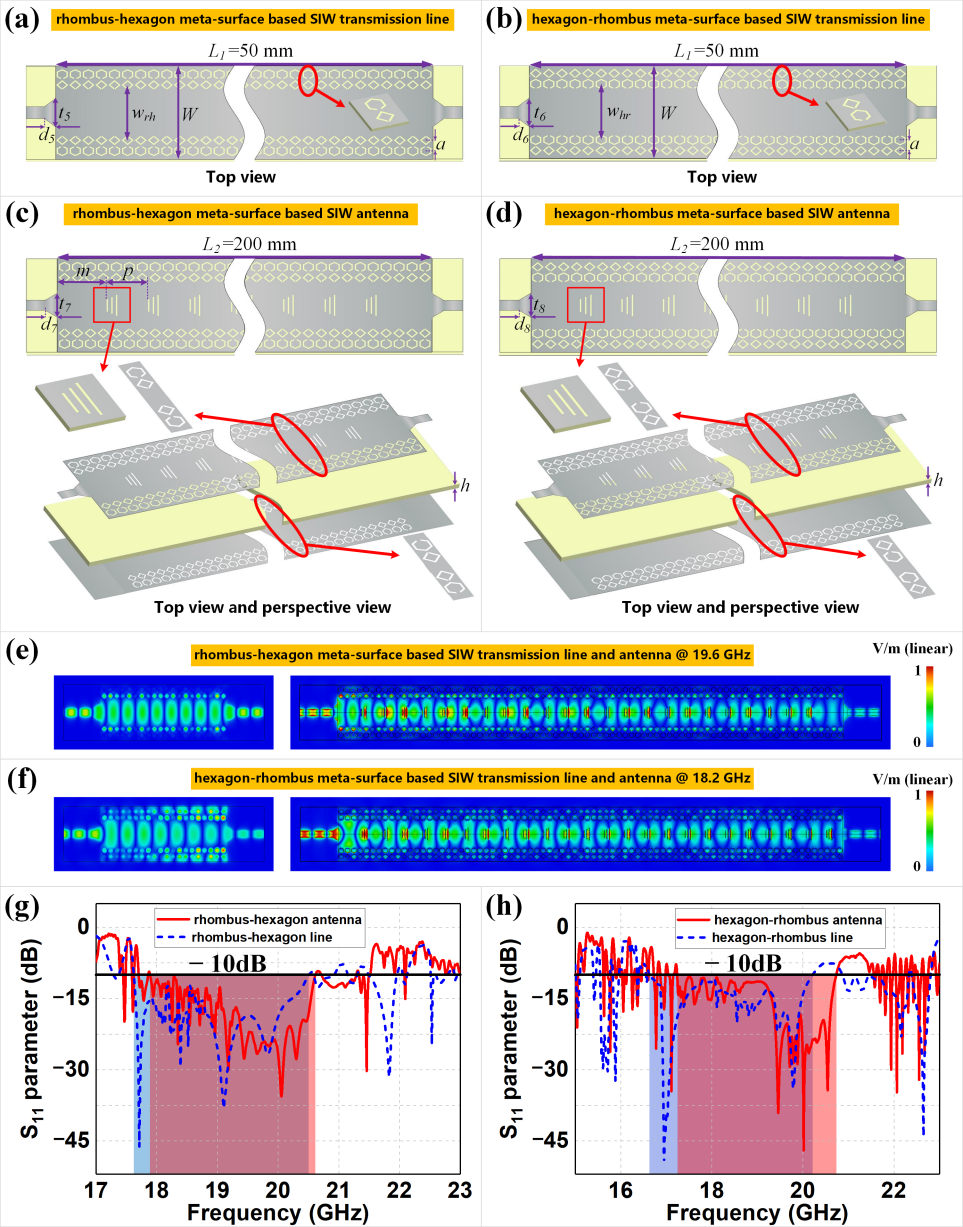

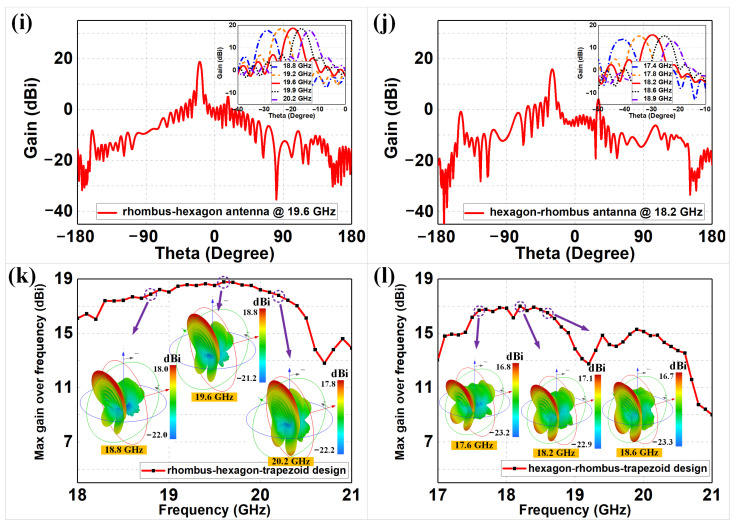
The coplanar (**a**) rhombus–hexagon and (**b**) hexagon–rhombus meta-surface-based SIW transmission lines, and the corresponding trapezoid-enabled meta-surface-based SIW leaky-wave antennas, as shown in (**c**,**d**). The geometric sizes are $L_{1}$ = 50 mm, *W* = 22 mm, $t_{5}$ = 6 mm, $d_{5}$ = 4 mm, $w_{rh}$ = 13 mm, $t_{6}$ = 6 mm, $d_{6}$ = 2.5 mm, $w_{hr}$ = 13.1 mm, $L_{2}$ = 200 mm, *m* = 9.4 mm, *p* = 8 mm, $t_{7}$ = 7 mm, $d_{7}$ = 1.8 mm, $t_{8}$ = 5 mm and $d_{8}$ = 2 mm. E-field distributions of the meta-surface-based SIW designs: (**e**) rhombus–hexagon and (**f**) hexagon–rhombus transmission lines and antennas. The corresponding reflection coefficients of the four SIW designs: (**g**) rhombus–hexagon and (**h**) hexagon–rhombus transmission lines and antennas. The far-field radiations of the meta-surface-based SIW leaky-wave antennas are demonstrated as follows: 2D patterns of (**i**) rhombus–hexagon and (**j**) hexagon–rhombus, and maximum gains of (**k**) rhombus–hexagon and (**l**) hexagon–rhombus with 3D patterns.

First of all, we would like to demonstrate the design method and logical relationship of all proposed coplanar SIW transmission lines and antennas that are constructed with meta-surfaces of sub-wavelength meta-atom arrays mentioned in Figure 1, which are summarized and listed in Table 1. One row of rhombus (or hexagon) units could construct one side of the virtual electric walls of basic meta-surface-based SIW transmission lines, as shown from Figure 2, Figure 3 and Figure 4. For the extended version of SIW lines, another row of meta-units is introduced to combine into rhombus–hexagon (or hexagon–rhombus) virtual electric walls, in order to obtain different bandwidth performances, as shown in Figure 5. Furthermore, all four proposed SIW transmission lines could be evolved into four SIW antennas through enabling trapezoid-shaped meta-surfaces, respectively. We now practically construct the basic version of coplanar SIW transmission lines and antennas with meta-surfaces, as shown in Figure 2. The double-layer rhombus (or hexagon)-shaped unit slots are etched on the top metal surface and bottom grounded plane instead of drilling the substrate, substituting for the metallized via holes of the standard SIW structure near the same locations and assembling the coplanar meta-surface-based SIW line, as shown in Figure 2a (or Figure 2b). The gaps of the double-layer ring slots are face-to-face on the top and in opposite directions on the bottom. The micro-strip feeding lines and transitions are attached to the SIW structure on the both ends. As the transmission lines or antennas exhibit periodic structures along the direction of wave propagation, we cut off the central parts of the SIWs, in order to better illustrate the detailed structure, in Figure 2. The width of the virtual electric walls from meta-surface-based SIW lines, expressed as $w_{r}$ (or $w_{h}$), is the key geometric dimension in the proposed design, where most of other parameters are related to it. Since the meta-surfaces would substitute the metallized via holes shown in the top areas of Figure 2a, the geometry sizes of the novel SIW structure, including $w_{r}$ or $w_{h}$, could be calculated and optimized from the classical method of the traditional SIW design. The equivalent width of the traditional SIW expressed as $w_{\mathrm{eff}}$ could be calculated as [50]

(5) $$w_{\mathrm{eff}}=w-1.08\frac{d^{2}}{s}+0.1\frac{d^{2}}{w}$$

where *w* refers to the physical width of the two rows of via holes, *d* is the diameter of a single hole, and *s* is the distance between neighboring holes. Thus, the parameter $w_{\mathrm{eff}}$ would determine the transmission characteristics and operating frequency [50], which would help us to determine the initial dimensions of $w_{r}$ and $w_{h}$, and we could further tune and optimize these values to fulfill the actual working frequency around 20 GHz. On this basis, the other parameter values could be determined within specific ranges. The *W* value is almost the same as the entire width of the traditional SIW, if choosing the same frame structure, material and working frequency. The *L* value depends on the actual requirements of the PCB board. Finally, the size of the transition structures, which are sensitive to the *S*-parameter, should be initialized and further optimized to satisfy the impedance matching requirements when we introduce the 50 Ω micro-strip lines at both ends [51,52,53], and such values should also be limited in the ranges smaller than $w_{r}$ and $w_{h}$.

Figure 2c,d further illustrate the rhombus and hexagon meta-surface-based SIW slot-antenna designs, respectively. The transverse multiple-slot meta-surfaces are introduced and periodically etched on the top plane of the SIW transmission lines, in order to construct the coplanar leaky-wave antennas with slow-wave structure. Such periodic profile modulation would introduce infinite space harmonics [54,55,56,57], and the phase constant $\beta_{n}$ of the *n*th space harmonic is calculated as

(6) $$\beta_{n}=\beta_{0}+\frac{2n\pi }{p}\;,n=0,\pm 1,\pm 2\ldots$$

where $\beta_{0}$ refers to the phase constant of the dominant mode, and *p* is the periodic length of the slot-units shown in Figure 2c. The space harmonic with phase constant $\beta_{-1}$ is usually chosen to be a fast mode, capable of converting transmission wave to radiation wave with backward beam angle as $\theta_{-1}=\sin^{-1}\left(\beta_{-1}/k_{0}\right)$, where $k_{0}$ is the wavenumber in free space, while the other harmonics remain in slow-wave mode and do not generate radiation. Therefore, these conditions could be quantified as $\beta_{0}>k_{0}$, $\left|\beta_{-1}\right|<k_{0}$ and $\left|\beta_{n}\right|>k_{0}$, where n<−1. Further, we can derive the range of *p* expressed as $2\pi /\left(\beta_{0}+k_{0}\right)<p<4\pi /\left(\beta_{0}+k_{0}\right)$ from these formulas to satisfy the condition that only n=−1 space harmonic can generate radiated fields. In addition, the minimum length of the slot can be calculated as $l=\lambda_{0}/\left(4\sqrt{\varepsilon_{r}}\right)$ [58]. Taking 20.0 GHz as an example, we could obtain $\beta_{0}$ = 601.57 rad/m, $k_{0}$ = 418.88 rad/m, $\beta_{-1}$ = −183.83 rad/m, $\beta_{-2}$ = −969.23 rad/m, *l* = 2.53 mm and 6.16 mm < *p* < 12.32 mm, which exactly meets the requirements of n=−1 mode radiation. At the beginning, the main single slot contributing the radiation is thus built using these calculated parameters, with dimensions of $l_{5}$ and $w_{3}$ in Figure 2c, as well as $l_{8}$ and $w_{3}$ in Figure 2d. Based on the single-slot structure, the updated unit consists of three transverse slots arranged in a trapezoid shape, which is proposed to construct the multiple-slot meta-surface and etch on the top plane of the SIW component. Inside each unit cell, the slot located in the end side with the longest length acts as the original main slot, and the extra two shorter slots that are linearly trapezoidal are designed to reduce undesired reflection, where the width gradient increases along the direction of the inside transmission wave. Since the parameter values or ranges, especially *l* and *p*, are calculated by classical theory, fulfilling the n=−1 space harmonic that adapts to a standard SIW structure with metallized via holes, we take them as the initial values or ranges and continue to optimize the geometric sizes of the main slot to satisfy the specific meta-surface-based designs, where the rest of the dimensions of the trapezoid unit would undergo synchronized optimization to hold the low reflections. All the corresponding geometric sizes in Figure 2 are labeled and listed in the figure and caption, and the sizes of the trapezoid units have a minor difference between the rhombus and hexagon meta-surface-based designs, in order to achieve their respective optimum performance. Compared to the conventional leaky-wave antenna with identical single slots, each unit of the trapezoid meta-surface in our proposed antennas enables local impedance transitions to suppress extra reflections, where all the slots are distributed with uniform aperture. There are also two common impedance gradient methods in the traditional SIW leaky-wave antenna designs, such as locating tapered increased single slots at the beginning [59,60] and periodically changing the slot length throughout the entire line [61,62]. Both of these design methods can achieve a significantly optimized $S_{11}$ level, while the overall length of the SIW structure cannot be arbitrarily set, due to the demands involved with adapting to a gradually changing geometry. Meanwhile, our proposed trapezoid meta-surface could also obtain low reflections, and the minimum changing step size is the small distance *p* of the trapezoid unit. As a result, the rhombus-, hexagon- and trapezoid-shaped meta-surfaces assembled in the SIWs all have a coplanar design and can be etched by copper cladding technology, which avoids the drilling of metallized via holes while realizing easy fabrication and significantly increasing device reliability.

### 2.3. Full-Wave Simulations

Full-wave simulations (CST Microwave Studio with Frequency Domain Solver) were performed to verify the coplanar SIW transmission lines and SIW antennas, as shown in Figure 3 and Figure 4, respectively. Figure 3a (or Figure 3b) demonstrates the two-dimensional E-field distributions on the cutting plane of *h*/2 for the rhombus (or hexagon) meta-surface-based SIW transmission line at 19.99 GHz (or 19.27 GHz). We can observe that the ${\mathrm{TE}}_{10}$ mode, the same as the rectangular waveguide, was successfully established, with minor leakage of the E-field outside the meta-surface rows. The E-field results prove that the two proposed meta-surface-based SIW lines can well imitate virtual electric walls and replace metallized via holes. The rhombus design demonstrates better electric wall performance with lower sidewall leakage of E-fields than the hexagon design. Figure 3c,d illustrate the corresponding *S*-parameters of the rhombus and hexagon SIW lines, respectively. We can observe that the rhombus design has $S_{11}$ values less than −10 dB ranging from 18.59 GHz to 20.37 GHz, with a minimum value of −38.37 dB at 19.99 GHz and relative bandwidth of 9.14%, while the hexagon design has $S_{11}$ values less than −10 dB ranging from 16.78 GHz to 20.68 GHz, with a minimum value of −27.47 dB at 19.27 GHz and relative bandwidth of 20.82%. We can also observe that there were some frequency shifts in the SIW lines and the original units, which can mainly be attributed to the fact that the unit cells were stimulated using ideal periodic Floquet models, while the meta-surfaces etched in practical structures would be affected by truncated boundary conditions. However, both the rhombus and hexagon SIW lines still achieved satisfactory power transfer with a lower reflection coefficient and wider bandwidth, respectively.

Figure 4a,b demonstrate the corresponding E-field distributions on the top plane of the rhombus and hexagon meta-surface-based SIW antennas at 19.60 GHz and 18.00 GHz, respectively. We can observe that the significant E-fields were distributed over the trapezoid-based meta-surfaces, which would excite high-gain radiations in the far-field. The field leakage at the sides still remained at a low level, as with the previous SIW lines. Figure 4c,d illustrate the corresponding *S*-parameters of the rhombus and hexagon SIW antennas with their comparison groups, respectively. In order to verify the effect of suppressing extra reflections from the trapezoid meta-surfaces, the traditional single-slot SIW antennas with the same external rhombus and hexagon meta-surface-based structures were also included in the $S_{11}$ simulations, for comparison purposes. Note that the proposed designs in Figure 4c,d and their comparison groups of single-slot structures should optimize the respective sizes of the microstrip transitions, achieving their best *S*-parameter performances. The −10 dB bandwidths of the trapezoid meta-surface designs are marked as red areas, while those of single-slot designs are marked as blue areas, and the intersections are marked as purple areas. We can observe that the trapezoid meta-surface designs had better reflection coefficients than the single-slot design, which proves that our trapezoid design enables better impedance matching through the transitions of the gradient slots. The rhombus–trapezoid design has $S_{11}$ values less than −10 dB ranging from 18.94 GHz to 21.09 GHz, with a minimum value of −50.04 dB at 19.31 GHz and a relative bandwidth of 10.74%, while the hexagon–trapezoid design has $S_{11}$ values less than −10 dB ranging from 17.20 GHz to 20.90 GHz, with a minimum value of −20.65 dB at 20.29 GHz and a relative bandwidth of 19.42%. We can also observe that the trapezoid meta-surface SIW antennas and the corresponding transmission lines showed similar trends in $S_{11}$, indicating that the introduction of a trapezoidal meta-surface only affects the reflection coefficient, to a certain extent.

Figure 4e–h continue to illustrate the two- and three-dimensional far-field radiation patterns of the coplanar trapezoid meta-surface-based SIW antennas, as well as the maximum gains over frequencies. We can observe that both the proposed designs generate highly directive fan beams and achieve satisfactory beam scanning within their operating frequency bands. For example, at the frequencies of 19.6 GHz and 18.0 GHz, the maximum gains of the rhombus and hexagon SIW antennas were 18.10 dBi and 17.00 dBi, respectively. The radiation efficiency and aperture efficiency thus could be obtained through comparing the gains to the directivity or aperture of the antenna. To be more specific, the radiation efficiency is calculated from the ratio of gain to directivity, expressed as $\eta_{ra}=G/D$, where the directivity can be obtained from the simulations [63,64,65]. Meanwhile, the aperture efficiency of the meta-surface-based SIW antenna can be calculated by $\eta_{ap}=\left(G\lambda^{2}\right)/\left(4\pi A\right)$, where *A* is the physical aperture of the antenna [66,67,68]. Therefore, the radiation efficiencies of the rhombus–trapezoid and hexagon–trapezoid SIW antennas are 87.10% and 81.28%, respectively, and the corresponding aperture efficiencies are 52.02% and 47.88%, respectively. The rhombus–trapezoid design has a 3-dB beamwidth of $4.7^{\circ }$ in the E-plane and $58.9^{\circ }$ in the H-plane, while the hexagon–trapezoid design has a 3-dB beamwidth of $5.4^{\circ }$ in the E-plane and $46.1^{\circ }$ in the H-plane. We can also observe that the beam-scanning performances of the two proposed antennas are well demonstrated when the operating frequency varies within certain ranges with high-gain performance. The rhombus–trapezoid meta-surface-based SIW antenna has scanning beams in $-24^{\circ }$, $-22^{\circ }$, $-20^{\circ }$, $-18^{\circ }$ and $-16^{\circ }$ at 19.2 GHz, 19.4 GHz, 19.6 GHz, 19.8 GHz and 20.0 GHz, respectively, while the hexagon–trapezoid design has scanning beams in $-42^{\circ }$, $-37^{\circ }$, $-32^{\circ }$, $-28^{\circ }$ and $-23^{\circ }$ at 17.2 GHz, 17.6 GHz, 18.0 GHz, 18.4 GHz and 18.8 GHz, respectively. Every 0.4 GHz frequency offset would result in $4^{\circ }$ beam steering for the rhombus antenna and $5^{\circ }$ beam steering for the hexagon antenna. Some of the three-dimensional radiation patterns are shown in Figure 4g,h. Both the rhombus–trapezoid- and hexagon–trapezoid-shaped antennas maintain high-gain radiations over the examined bandwidth, from 19.0 GHz to 20 GHz and from 17.0 GHz to 18.8 GHz, respectively. Although there are some fluctuations in the maximum gains, this is reasonable, as the electromagnetic responses from the meta-surfaces vary with frequency.

### 2.4. Extended SIW Design and Simulations

We extend our design to coplanar two-row based meta-surfaces by combining rhombus and hexagon structures in different orders, based on the original transmission lines and antennas in Figure 2. Figure 5a demonstrates the rhombus–hexagon meta-surface-based SIW transmission lines, where the rows of hexagon unit cells are etched at one cell spacing outside the rhombus cell rows of the original rhombus meta-surface-based SIW line. Similarly, Figure 5b demonstrates the opposite structure to Figure 5a, with hexagon rows inside and rhombus rows outside. Moreover, the same trapezoid-shaped meta-surfaces are also introduced to the two mixed SIW transmission lines, thus constructing the complex coplanar meta-surface combined SIW antennas shown in Figure 5c,d. Based on the optimization process of the original meta-surface-based SIWs, the four proposed designs in Figure 5 could use the optimized results from Figure 2 as initial values and further simulate and adjust specific geometric sizes such as transition sizes to obtain low reflections. The corresponding E-field distributions and $S_{11}$ parameters of the four proposed designs are illustrated in Figure 5e–h. We can observe, from the E-fields, that good ${\mathrm{TE}}_{10}$ mode transmission is achieved in each of the transmission lines and antennas, with less edge leakage compared to that shown in Figure 3 and Figure 4. The −10 dB bandwidths of the meta-surface-based antennas are marked as red areas, while those of transmission lines are marked as blue areas, and the intersections are marked as purple areas. The rhombus–hexagon transmission line in Figure 5a has $S_{11}$ values less than −10 dB ranging from 17.62 GHz to 20.50 GHz, with a minimum value of −46.22 dB at 17.71 GHz and a relative bandwidth of 15.11%, while the hexagon–rhombus transmission line in Figure 5b has $S_{11}$ values less than −10 dB ranging from 16.63 GHz to 20.21 GHz, with a minimum value of −49.39 dB at 16.95 GHz and a relative bandwidth of 19.44%. For the complex SIW antennas in Figure 5c,d, the corresponding ranges are from 17.89 GHz to 20.61 GHz with a minimum of −35.61 dB at 20.05 GHz and relative bandwidth of 14.13%, and from 17.24 GHz to 20.73 GHz with a minimum of −46.99 dB at 20.02 GHz and relative bandwidth of 18.38%. The original rhombus structures in Figure 2a,c concentrate on obtaining lower reflections, while the new rhombus–hexagon designs in Figure 5a,c increase the bandwidth on this basis. Similarly, the original hexagon structures in Figure 2b,d have significantly wider bands with acceptable reflections, while the hexagon–rhombus designs in Figure 5b,d retain these broad bands and achieve greatly reduced reflections at specific frequencies. These different results are mainly attributed to the primary shielding effect of the inside meta-surfaces and the secondary shielding effect of the outside meta-surfaces, where the primary meta-unit rows determine the overall transmission performance and the secondary cells fine-tune the reflections on this basis. As a result, all of the proposed devices obtain good reflection and bandwidth, but with different priority.

For the complex coplanar meta-surface combined SIW antennas, the far-field radiation patterns, including the beam-scanning performance, are demonstrated in Figure 5i–l. We can observe that both the proposed antennas in Figure 5 obtained similar high-gain fan beams and satisfactory beam scanning, compared to the originals in Figure 4. For example, at 19.6 GHz and 18.2 GHz, the maximum gains of the two coplanar SIW antennas were 18.8 dBi and 17.1 dBi, respectively. Based on the same calculation method for the rhombus and hexagon antennas from Figure 3, the radiation efficiencies of the rhombus–hexagon and hexagon–rhombus meta-surface-based SIW antennas are 91.20% and 83.18%, respectively. Moreover, the corresponding aperture efficiencies are 61.12% and 47.92%, respectively. The other radiation results of the SIW antennas from Figure 5c,d, including the 3-dB beamwidth on the E- and H-plane, beam scanning angles at specific frequencies and beam scanning rate, are all summarized and listed in Table 2. We can conclude that the extended SIW transmission lines and antennas in Figure 5 are still capable of propagating the electromagnetic fields well and generating high-gain beam-scanning fan beams. Moreover, every 0.4 GHz frequency offset resulted in approximately $4^{\circ }$ and $5^{\circ }$ beam steering for the proposed meta-surface-based SIW antennas. Moreover, the *S*-parameter results appear more flexible and tunable, compared to those of the original devices. The complex coplanar meta-surface-based designs provide promising candidates for building novel electromagnetic devices on PCB-based systems. The four SIWs can flexibly handle different bandwidth requirements, where the length value *L* of the two antennas could be freely adjusted with a minimum step value of *p*, instead of a larger length, for impedance matching with traditional single-slot designs.

## 3. Discussion of Experimental Results

**Figure 6:**
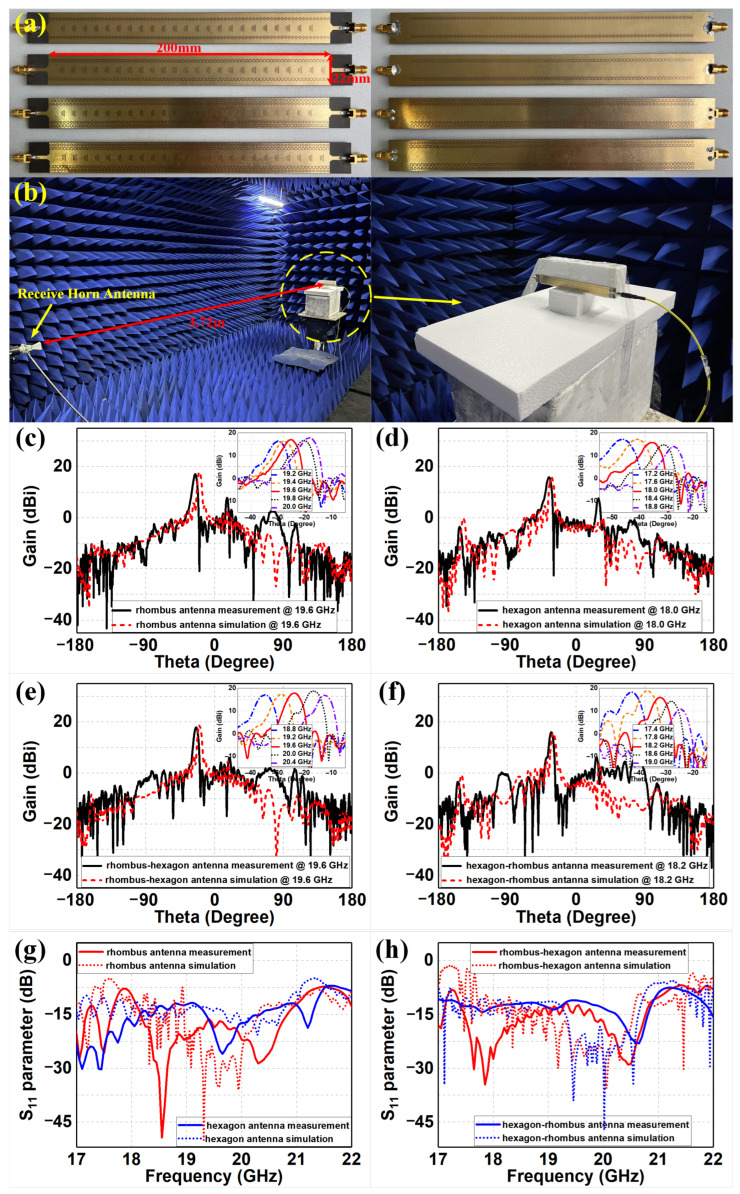
Photos and experimental results of the manufactured meta-surface-based SIW leaky-wave antennas with comparison to the corresponding simulations. (**a**) Photos of the four SIW antennas. (**b**) The corresponding experiments in a microwave chamber. The measured radiation patterns of the (**c**) rhombus, (**d**) hexagon, (**e**) rhombus–hexagon and (**f**) hexagon–rhombus meta-surface-based SIW leaky-wave antennas, The measured reflection coefficients of the (**g**) rhombus and hexagon designs, and (**h**) rhombus–hexagon and hexagon–rhombus designs.

Finally, we fabricated the coplanar rhombus, hexagon, rhombus–hexagon and hexagon–rhombus meta-surface-based SIW leaky-wave antennas and carried out corresponding experiments in a microwave chamber to verify the proposed designs, as shown in Figure 6a,b. Through simple copper cladding and etching technology, the meta-surfaces and microstrip structures were printed on both sides of the substrate slabs. In addition, standard SMA connectors were soldered on both ends of the substrates for feeding excitation. The four meta-surface-based SIW antennas under test with equal height to the receiving antenna were set as transmit antennas and properly assembled on the center position of the turntable. A vector network analyzer was applied for measuring the reflection coefficients of the proposed antennas, and a standard diagonal horn antenna with the distance of 3.72 m to the center of the turntable was utilized to receive the electromagnetic fields and measure the radiation performance. The experimental far-field radiation results of four proposed SIW antennas are demonstrated in Figure 6c–f, with a comparison to the corresponding simulations. We can observe that all proposed antennas demonstrated highly directive radiation and beam-scanning performance, as designed. The measured gain values of the center frequencies were 17.06 dBi, 15.79 dBi, 17.9 dBi and 15.97 dBi at 19.6 GHz, 18 GHz, 19.6 GHz and 18.2 GHz, respectively, as shown in Figure 6c–f. Based on these gain results, we could calculate the measured radiation efficiencies of the rhombus, hexagon, rhombus–hexagon and hexagon–rhombus meta-surface-based SIW antennas as 68.55%, 61.52%, 74.13% and 64.12%, and the measured aperture efficiencies were 40.94%, 36.23%, 49.68% and 36.94%, using the same methods as those for calculating the simulated radiation and aperture efficiencies. The measured efficiencies are approximately 10–18% lower than the simulated efficiencies. The corresponding beam-scanning measurements are also illustrated, with nearly the same frequency ranges as in Figure 4 and Figure 5. The corresponding $S_{11}$ results are shown in Figure 6g,h with a comparison to the corresponding simulations, where the trends of the *S*-parameter curves are similar to those in the original simulations, achieving their different bandwidth design aims. For Figure 6g, the rhombus SIW antenna has $S_{11}$ values less than −10 dB ranging from 18.05 GHz to 21.1 GHz, with a minimum value of −49.35 dB at 18.55 GHz and relative bandwidth of 15.58%. Meanwhile, the hexagon SIW antenna has $S_{11}$ values less than −10 dB ranging from 16.20 GHz to 21.40 GHz, with a minimum value of −30.33 dB at 17.45 GHz and relative bandwidth of 27.66%. For Figure 6h, the rhombus–hexagon SIW antenna has $S_{11}$ values less than −10 dB ranging from 17.35 GHz to 21.00 GHz, with a minimum value of −34.57 dB at 17.85 GHz and relative bandwidth of 19.04%. Meanwhile, the hexagon–rhombus SIW antenna has $S_{11}$ values less than −10 dB ranging from 16.80 GHz to 20.95 GHz, with a minimum value of −23.04 dB at 20.65 GHz and relative bandwidth of 21.99%. We can observe that the measured gains and *S*-parameters were mostly in agreement with the simulations, with only a few degradations or frequency shifts. As a result, there are some discrepancies in the simulated and measured results from the proposed antennas. These degradations were mainly attributed to the fabrication tolerance of the meta-surfaces, the welding quality of the SMA connectors and the measurement deviations. The fabrication tolerance could affect the geometric sizes and substrate electromagnetic properties of the unit cells, which would result in discrepancies in the electromagnetic response from the meta-surfaces. The welding quality would affect the transmission characteristics of the SMAs, which in turn affects the radiation performance of the antennas. Moreover, the potential antenna deformation during the measurement and the effects of fixed brackets on electromagnetic waves could also lead to measurement deviations. However, all four of the proposed meta-surface-based SIW antennas demonstrated their beam-scanning ability, with flexible reflection coefficients and bandwidths.

## 4. Conclusions

In conclusion, we introduced coplanar rhombus- and hexagon-shaped meta-surfaces into the design of SIW transmission lines, and constructed four associated coplanar meta-surface-based SIW antennas through the application of trapezoid-shaped meta-surfaces. The four antennas obtain maximum gains of 18.1 dBi, 17.0 dBi, 18.8 dBi and 17.1 dBi, where the simulated radiation efficiencies are 87.10%, 81.28%, 91.20% and 83.18%, and the simulated aperture efficiencies are 52.02%, 47.88%, 61.12% and 47.92%. By replacing metallized via holes with specifically modified meta-surfaces, all of the proposed designs were shown to possess more flexible and tunable reflection coefficients and bandwidth performances, where the relative bandwidths thus are 10.74%, 19.42%, 14.13% and 18.38%. Further, the four coplanar meta-surface-based SIW antennas presented highly directive beam-scanning fan beams at K-bands with beam-scanning rate $4^{\circ }$ (rhombus and rhombus–hexagon designs) and $5^{\circ }$ (hexagon and hexagon–rhombus designs) per 0.4 GHz, paving the way for the development of mm-Wave PCB-integrated antennas or sensors with freely customizable electrical performance.

## Figures and Tables

**Table 1:** The design method and logical relationship of all proposed coplanar meta-surface-based SIW transmission lines and antennas.

	Basic Meta-Surface-Based Design		Extended Meta-Surface-Based Design	
	Figure 2, Figure 3 and Figure 4		Figure 5	
Transmission line	Rhombus row	Hexagon row	Rhombus–hexagon rows	Hexagon-rhombus rows
Antenna	Rhombus row	Hexagon row	Rhombus–hexagon rows	Hexagon–rhombus rows
	(Trapezoid enabled)	(Trapezoid enabled)	(Trapezoid enabled)	(Trapezoid enabled)

**Table 2:** Comparison of radiation performance between rhombus–hexagon and hexagon–rhombus meta-surface-based SIW antennas.

Radiation Performance	Rhombus–Hexagon Meta-Surface-Based SIW Antenna	Hexagon–Rhombus Meta-Surface-Based SIW Antenna
3-dB beamwidth on E-plane	$4.7^{\circ }$	$5.2^{\circ }$
3-dB beamwidth on H-plane	$60.1^{\circ }$	$49.3^{\circ }$
Beam-scanning angles @ frequency	$-29^{\circ }$ @ 18.8 GHz	$-41^{\circ }$ @ 17.4 GHz
	$-24^{\circ }$ @ 19.2 GHz	$-35^{\circ }$ @ 17.8 GHz
	$-20^{\circ }$ @ 19.6 GHz	$-30^{\circ }$ @ 18.2 GHz
	$-16^{\circ }$ @ 19.9 GHz	$-25^{\circ }$ @ 18.6 GHz
	$-13^{\circ }$ @ 20.2 GHz	$-23^{\circ }$ @ 18.9 GHz
Beam-scanning rate	$4^{\circ }$/0.4 GHz	$5^{\circ }$/0.4 GHz

## Data Availability

Not applicable.
